# Editorial for the Special Issue “Cutaneous Biology, Molecular Dermatology and Dermatopathology”

**DOI:** 10.3390/ijms26062694

**Published:** 2025-03-17

**Authors:** Bijan Safai

**Affiliations:** 1 Department of Dermatology, New York Medical College, Valhalla, NY 10595, USA; bijan_safai@nymc.edu; 2 Dermatology Department, NYC HHC/Metropolitan Hospital Center, New York, NY 10029, USA

## 1. Introduction

Interest in molecular technology continues to grow at a notable rate, particularly within the realm of health sciences. Although initial emphasis was placed on hematology and oncology, the scope of molecular research has progressively expanded to include dermatology. Advancements in this specialty facilitate a deeper understanding of disease pathophysiology, sparking further innovations in both diagnostic and therapeutic approaches. Recent preferences in diagnostic procedures and individualized treatment planning have leaned towards DNA or RNA-based molecular techniques, such as immunohistochemistry, in situ hybridization, PCR, and sequencing. This shift is particularly notable following the authorization of BRAF/MEK inhibitors for treating metastatic melanoma, where detecting BRAF mutations in affected patients proves critical. Such targeted treatments are also becoming integral in managing inflammatory skin conditions, including psoriasis, atopic dermatitis, and vitiligo, as well as in dermatological oncology [1]. In the context of wound care, cellular therapies have emerged prominently. Chronic wounds have shown rapid and superior healing using mesenchymal stromal cells and pluripotent stem cells, compared to traditional treatments. Moreover, innovative approaches such as protein replacement, gene therapy, and gene editing have recently been applied with success in treating genodermatoses. This Special Issue delves into the latest research within cutaneous biology, molecular dermatology, and dermatopathology, shedding light on molecular advancements that have recently garnered significant interest and promise substantial development [2].

## 2. An Overview of Published Articles

Ketoprofen is one of the most common pharmaceuticals that can cause skin eruptions in sun-exposed areas. It is widely used both orally and topically in the treatment of several musculoskeletal and joint disorders, and symptomatic treatment of pain and fever. Ketoprofen can cause both photoallergic and phototoxic skin reactions. Although the benzophenone group in its photoallergic chemical structure may play a role in photoallergic reactions, the exact mechanism behind phototoxic reactions against ketoprofen has yet to be determined. The idea of this paper was to determine the ketoprofen phototoxicity in the epidermis in different environments. They analyzed the cytotoxic and phototoxic activity of ketoprofen towards human melanocytes and fibroblasts in vitro. Ketoprofen in combination with UVA radiation induced apoptotic cell death, manifested by disruption of thiol levels, and decreased mitochondrial membrane potential, cell cycle disruption, DNA fragmentation, and activation of proteins related to cell death, such as p53 and cytochrome c. The results indicate that the highest risk of photosensitivity reactions occurs after direct contact with the drug and UV irradiation. These findings may help avoid adverse effects of ketoprofen related to phototoxicity [3].

The idea of the second paper was to utilize liver fluke (*O. felineus*) excretory-secretory products (ESP) and inactivated cells to promote wound healing in diabetic mice. In this 14-day experiment, results showed the significant wound healing potential of these products, and these results were comparable to commercially available topical PDGF products. The authors looked at histologic and biochemical indicators to assess and compare the wound healing in the vehicle-only group, the PDGF applied group, ESP group, and inactivated egg group. The ESP and inactivated egg products of liver fluke mediated canonical wound-healing processes, including macrophage phenotype switching (M1-to-M2), angiogenesis, and extracellular matrix reorganization. *O. felineus* eggs, the isolation of proteins common to major fractions (ESP, lysate, and eggs), and the identification of other bioactive molecules (including microRNAs), followed by in vitro and in vivo testing, offer promising avenues for the discovery of novel wound-healing agents for chronic non-healing wounds [4].

Terlikowska-Brzósko et al. evaluated new biomarkers to differentiate psoriasis vulgaris and atopic dermatitis. Specifically, they focused on two major biomarkers: TPP2 (tripeptidyl peptidase 2), a subunit of 20S immunoproteasome complex, and PSMB8 (proteasome subunit beta type-8), a protease involved in the proteasome-ubiquitin pathway in MHC class I antigen presentation. They studied TPP2 mRNA and PSMB8 mRNA levels in addition to human β-defensin-2 (hBD2) and involucrin (IVL) levels. The expression of TPP2 mRNA was inversely associated with the diagnosis of atopic dermatitis. The PSMB8 mRNA levels were inversely associated with psoriasis vulgaris. hBD-2 mRNA results alone were sufficient to exclude healthy skin biopsies from inflammatory lesions. PSMB8 mRNA from atopic dermatitis biopsies correlated positively with the body surface area. On the contrary, there was an inverse correlation between IVL mRNA and skin itch intensity only in AD patients. They also created a diagnostic algorithm combining the biomarkers of hBD-2 mRNA, IVL, IVL mRNA, and PSMB8 mRNA by using artificial intelligence [5].

Arsenic is a hazardous environmental factor that poses significant risks to human health. Studies have revealed that arsenic (NaAsO_2_) critically undermines the functional integrity of dermal fibroblasts, constraining their viability, migration, and proliferative capacity [6]. Furthermore, it has been observed that arsenic binds to estrogen—a hormone known for its anti-inflammatory attributes that facilitate normal wound healing and regulate the over-accumulation of neutrophils at wound sites [7]. Despite these findings, the effects of arsenic exposure on wound healing have not been elucidated in vivo. Dresler et al. examined the influence of arsenic on metabolic activity, viability, and gene expression by subjecting cultured dermal fibroblasts to arsenic exposure. Furthermore, the impact of inorganic arsenic exposure on key components of wound healing was investigated utilizing a mouse full-thickness wound model. It has been found that arsenic exposure in cultured fibroblasts prompts an augmentation in matrix metalloproteinase-1 (MMP1) expression, diminishes scratch closure, alters fibroblast morphology from a spindle to a round shape, suppresses fibroblast proliferation, and hinders metabolism, leading to a reduction in cell viability. In vivo exposure to 10 µM NaAsO_2_ in drinking water for eight weeks inhibited wound closure and elevated wound erythema levels, particularly in female mice, whereas these effects were not observed in male mice. Gene expression analysis of skin from the wound area revealed increased expression of Arsenic 3-Methyltransferase (As3mt) and Estrogen Receptor 2 (Esr2) mRNA in female mice. The research indicated that NaAsO_2_ exposure disrupts normal wound healing mechanisms, as revealed by both in vitro and in vivo experimental trials. These findings suggest that individuals suffering from delayed wound closure and/or chronic non-healing cutaneous wounds could be at heightened risk in instances of prolonged exposure to arsenic [8].

Wound healing represents a complex biological process involving inflammatory cells, keratinocytes, endothelial cells, fibroblasts, and the cytokines they produce. Recent increases in the prevalence of chronic wounds have been attributed to rising instances of diabetes, an aging population, and disparities in healthcare access. These chronic wounds significantly enhance the risk of infection, prolong hospitalizations, may lead to amputation, and adversely affect patient quality of life, thereby escalating healthcare costs. Consequently, research into wound healing mechanisms and treatments has accelerated notably [9]. Within this domain, the application of stem cell therapy holds considerable promise for the management of chronic wounds. Stem cells support the healing process by enhancing regeneration and providing paracrine signaling molecules. The systematic review by Farabi and colleagues, which synthesized findings from 44 studies, has highlighted the utility of mesenchymal stem cells, particularly those sourced from adipose tissue, for their ease of extraction, plentiful availability, and potent pro-angiogenic qualities. These cells have proven effective in treating conditions such as peripheral arterial disease, diabetic ulcers, hypertensive ulcers, and wounds incurred post-Mohs micrographic surgery [9].

As outlined in the systematic review, stem cell therapy exerts a significant therapeutic effect by differentiating into various cellular components of the wound matrix, secreting growth factors, and catalyzing angiogenesis. The clinical implementation of stem cell therapy not only promises to enhance patient outcomes and quality of life but also to diminish healthcare expenditures. The growing evidence supports a broader adoption and integration of stem cell interventions in clinical practice to address complex wound management challenges effectively [9].

Innate lymphoid cells (ILCs) are integral to the innate immune system, playing crucial roles in host immune responses, tissue homeostasis, and the regulation of epithelial and adaptive immune functions. ILCs are categorized as natural killers (NKs), ILC1, ILC2, ILC3, and ILCreg, based on their transcription factors and cytokine profiles [10]. ILC3s are distinct in expressing CD117, distinguishing them from other ILCs, and are essential for maintaining skin homeostasis, mediating inflammation, and responding to infections. Despite their importance, the precise mechanisms by which ILC3s function in skin biology and disease remain poorly understood. The review by To et al. synthesizes current evidence on the mechanisms of ILC3 action and their connection to skin diseases while identifying knowledge gaps to guide future research [11]. This comprehensive review highlights that ILC3 influences the function of sebaceous glands and the balance of commensal bacteria, suggesting a potential role in acne pathogenesis through interactions with Cutibacterium acnes, which utilize sebum as an energy source. While the role of ILC3 has been established in conditions such as alopecia areata, hidradenitis suppurativa, psoriasis, and atopic dermatitis, its involvement in acne remains largely unknown. It is hypothesized that ILC3 interacts with C. acnes and affects macrophages involved in inflammatory acne, potentially contributing to acne pathogenesis. Additionally, chronic ultraviolet light exposure has been noted to stimulate keratinocytes to increase IL-17 and IL-22 production via ILC3, triggering inflammation, macrophage recruitment, and wound healing. However, the exact mechanisms underlying these processes remain to be elucidated. Although the relationship between ILC3 and immune cells such as neutrophils, dendritic cells, and T and B cells has been described in the intestines and tonsils, these interactions have not yet been clarified within the skin environment or in relation to the cutaneous microbiota [11]. ILCs are an emerging topic in the literature, and further exploration of ILC3 mechanisms and their relationship with the microbiota holds significant promise for addressing the uncertainties surrounding the pathogenesis and treatment of skin diseases.

The comprehensive review by D’Arino et al. [12] explores the impact of skin aging on the pathogenesis of skin cancer. Skin aging is a natural process influenced by both internal and external factors. External factors are primarily lifestyle-related, with UV radiation being the most significant. Internal factors include a decline in the cell’s repair capacity over time, leading to the accumulation of damaged senescent cells, the formation of a senescence-associated secretory phenotype, telomere shortening, and age-related inflammation. While cellular aging is linked to cancer due to increased damage, it also has cancer-preventive effects through paracrine secretions that reduce the proliferative capacity of cells [12]. The tumor microenvironment plays a crucial role in enabling tumor cells to grow, evade immune cells, and metastasize. The cancer stroma comprises fibroblasts or mesenchymal cells, known as cancer-associated fibroblasts (CAFs). Recent studies indicate that skin aging creates a pro-tumorigenic environment. This is facilitated by increased oxidative stress, reduced antioxidant defenses, and heightened mutations in aged cells. However, the decreased or halted proliferation in an aging-associated environment can also inhibit tumor formation. Notably, long telomeres, which shorten with aging, have been linked to melanoma and non-melanoma skin cancers. The addition of external factors such as UV radiation exacerbates DNA damage and oxidative stress, compounding the aging-related effects from internal factors, thus contributing to a pro-tumorigenic environment. Additionally, GDF15, associated with aging and a marker of premature aging, is overexpressed in melanoma patients. Conversely, IGF1 levels decrease with aging, reducing the adhesion, proliferation, and oxidative stress resistance of keratinocytes. In the immune microenvironment, a condition known as inflammaging occurs, characterized by the continuous stimulation of the innate immune response and impairment of the adaptive immune response due to proinflammatory cytokines secreted by aging cells. During aging, mitochondrial function is compromised due to an increase in reactive oxygen species, which also impacts glucose metabolism. The post-translational modification of proteins through O-GlcNAcylation increases with heightened glycolysis. O-GlcNAcylation supports tumor formation by promoting immunosuppression within the tumor microenvironment [12].

In conclusion, this thorough review highlights the importance of understanding and elucidating the mechanisms of skin aging as a critical step in addressing the formation and progression of skin cancer. Slowing the aging process—particularly by minimizing external contributing factors—may serve as an effective strategy for preventing the development and progression of skin cancer [12].

## 3. Conclusions

This Special Issue, focused on cutaneous biology, molecular dermatology, and dermatopathology, features seven carefully selected educational papers that delve into the intricate cellular and molecular mechanisms underlying these topics. Additionally, these contributions highlight the vast potential for further exploration and discovery in the field of skin biology. As we look to the future, advancements in molecular techniques—such as single-cell genomics, spatial transcriptomics, and artificial intelligence—are anticipated to propel groundbreaking research and innovations in skin biology.
